# Modern Wood-Based Materials for Sustainable Building

**DOI:** 10.3390/ma19122620

**Published:** 2026-06-18

**Authors:** Dorota Dukarska, Jakub Kawalerczyk

**Affiliations:** Department of Mechanical Wood Technology, Faculty of Forestry and Wood Technology, Poznań University of Life Sciences, Wojska Polskiego 38/42, 60-627 Poznań, Poland; jakub.kawalerczyk@up.poznan.pl

## 1. Introduction

The contemporary use of wood in construction is undergoing a significant transformation driven not only by changes in structural design approaches but, above all, by the development of wood-based materials, which represent a processed and improved form of wood [1]. This is accompanied by a growing interest in wood construction as it is a more environmentally friendly solution that aligns with the principles of sustainable development. Thanks to their properties, wood and wood-based materials are increasingly seen as alternatives to high-carbon building materials, helping to reduce the carbon footprint and promote energy-efficient construction [2,3,4]. Research conducted since the early 20th century has led to a systematic and ever-improving understanding of wood’s properties, enabling its rational use in the context of modern design and operational requirements. These changes have resulted in the development of modern timber construction, which relies heavily on new-generation wood-based materials, prompting the search for new material and technological solutions aimed at ensuring the efficient and responsible use of resources. Increasing importance is placed not only on the rational management of timber resources, but also on the use of new alternative raw materials and the implementation of circular economy principles and innovative technologies such as 3D printing [5,6,7,8]. For these reasons, interest in this subject has markedly increased among the scientific community in recent years, as reflected in the papers published in this Special Issue, which aimed to present the latest knowledge and current trends in the application of innovative solutions that can be used in modern wood construction. It is worth emphasizing the interdisciplinary nature of the published works, evident in the combination of various fields of science, such as materials engineering, wood technology, forestry, and ecology. This synergy constitutes a significant value of this series, highlighting the potential to utilize the proposed solutions in developing modern and eco-friendly construction, with particular emphasis on timber construction.

## 2. Overview of Contributions to This Special Issue

This article summarizes the published research results, which can be divided into the following thematic areas: 1—expanding the raw material base through using alternative wood-based raw materials; 2—applying the principles of the circular economy through using non-wood biomass, industrial waste (including biowaste), and recycled materials; 3—advanced composite and low-emission materials; and 4—using advanced technologies for raw material modification and the production of wood-based materials.

### 2.1. Alternative Wood-Based Raw Materials

Intensive research has been conducted for many years in the search for alternative wood raw materials and ways to use them effectively in the wood-based materials industry. Particular attention has been paid to fast-growing species, such as poplar [9], paulownia [10], and energy willow [11], as well as juvenile wood [12], whose specific properties can influence the characteristics of the finished materials. Further research provides important information regarding the technological suitability of these raw materials and their potential applications in the wood industry. One important article in this SI is that by Wang et al. [13] on the use of trembling aspen (*Populus tremuloides*), a dominant deciduous tree species in North America, in the production of engineered wood products (EWPs), which expands the potential applications of this raw material. According to the authors, the industrial uses of trembling aspen are currently limited to producing pulp, paper, and wood-based composite boards. Its oven-dried (SG) value is 0.4, which is approx. 3.5% higher than that reported in the basic literature. The authors classified trembling aspen lumber into two grades, SS Grade and No. 2 Grade, and conducted wood testing using the MSR (Machine Stress-Rated) method, longitudinal stress wave (LSW), edgewise third-point bending (EWB), and ultimate tensile strength (UTS) testing. On this basis, it has been demonstrated that knot size is a major factor determining the properties of sawn timber, as increasing the maximum knot size by a half-inch (from one-quarter of an inch) results in a decrease in the minimum modulus of elasticity (MOE) of approx. 8.8% and a reduction in the UTS by 20.1%. They also found that about 44% of the trembling aspen lumber tested met the grade 1450f-1.3E requirements for MSR lumber, while 62% fell under grade 1200f-1.2E. There is therefore significant potential for using this wood species to produce E3-grade CLT panels, with a rejection rate of approx. 29%. Nevertheless, the strength parameters of this species were lower than those of spruce–pine–fir lumber. For the SS class, UTS values were 25.5% lower, and MOE was 11.3% lower. Based on this, it can be concluded that trembling aspen has significant potential for use in civil engineering, and the authors recommend that knot size be the primary criterion for sorting this raw material. The conducted research thus points to the better utilization of surplus hardwood resources through creating high-value-added products.

Czarnecki et al. [14] presented another solution to the growing shortages of wood raw materials in the wood-based board industry by analyzing the possibility of using juvenile birch and pine wood to produce particleboard with a density reduced to 550 kg/m^3^. The study evaluated the effect of this raw material on the physical and mechanical properties of the obtained particleboards. The boards were manufactured using branch wood from Scots pine (*Pinus sylvestris* L.) and silver birch (*Betula pendula Roth*), with 5 to 13 annual rings, ensuring that the material consisted exclusively of juvenile wood. The authors found that boards made from juvenile pine showed a higher internal bond (IB) than those made from birch, which resulted from their greater elasticity and better flexibility during pressing. However, boards made of juvenile pine had a modulus of elasticity (MOE) that was approximately 40% lower than that of birch boards, which, in turn, exhibited modulus of rupture (MOR) and modulus of elasticity (MOE) values comparable to those of industrially manufactured particleboard. In addition, boards made from juvenile wood exhibited weaker water resistance, as evidenced by increased thickness swelling (TS) and water absorption (WA) compared to boards made from mature wood, with pine boards showing poorer performance in this regard. In conclusion, the authors stated that juvenile birch is a more promising raw material than pine, offering adequate bending strength and satisfactory stiffness. Despite certain limitations resulting from their lower water resistance, all the boards produced met the requirements of the EN 312 standard for general-purpose boards (type P1). This suggests that juvenile wood can be a cost-effective and sustainable solution in producing boards intended, among other things, for furniture manufacturing or sandwich panels.

The search for alternative sources of wood and ways to reduce pressure on traditional forest resources has also contributed to growing interest in wood from plantation sources. Żabowski et al. [15] investigated the potential for upcycling biomass from 2-to-3-year-old plantation willow (*Salix viminalis* L.) and 5-year-old poplar (*Populus* spp.) to produce particleboard. These raw materials, known for their rapid growth, are typically used for energy purposes; however, the authors decided to investigate their potential as construction and furniture materials. Boards were manufactured with 5%, 10%, 25%, 50%, and 100% willow and poplar particle contents. The tests showed that boards made only from willow and poplar particles had MOR values approx. 49% higher than those of the reference boards. For poplar particleboard, an MOR value of 20 N/mm^2^ was obtained, which represented a significant improvement over the reference boards, for which this parameter was approx. 14 N/mm^2^. At the same time, these boards exhibited an IB value nearly three times higher than that of the reference material. Furthermore, the use of these wood species contributed to improved screw withdrawal (SWR) and water resistance. In terms of TS, the willow particleboard met the requirements of the standard for P3-type boards. According to the authors, willow- and poplar-based boards are promising and eco-friendly alternatives for the construction and furniture industries, while also contributing to resource efficiency and the reduction of carbon dioxide emissions.

Diversifying the raw materials used in particleboard production is also the subject of research by Wronka et al. (contribution 1), who analyzed the possibility of substituting traditional pine particles with hazel wood (*Corylus avellana* L.) in various proportions, i.e., 5%, 10%, 25%, 50%, and 100%. A positive effect of this substitution was observed on IB strength and SWR, whose values increased as the content of hazel particles increased; however, as the hazel content increased, the MOR and MOE values of the boards decreased. Despite this, most of the analyzed variants still met the requirements of EN 312 for P3 and P5 boards, which was attributed to the higher density of hazel wood compared to pine wood. At the same time, increasing the proportion of this raw material had a negative impact on the water resistance of the boards, particularly in variants containing a higher proportion of hazel wood with bark, evidenced by increased WA and TS. Particularly unfavorable results in this area were obtained for boards made exclusively from hazel wood, whose parameters exceeded the permissible values required by the EN 312 standard for P3–P7 boards. The authors noted that hazel wood can serve as a valuable substitute for pine wood, with 50% content of this raw material deemed the most advantageous, as it allows the boards to retain their desired properties while increasing the use of alternative raw materials. According to Wronka et al. (contribution 1), hazel wood can be used for the industrial-scale production of particleboard, provided that parameters related to moisture resistance are controlled.

### 2.2. Circular Economy Approaches in the Utilization of Non-Wood Biomass and Industrial Waste

Another issue addressed in this Special Issue was implementing circular economy principles and expanding the raw material base used for the production of lignocellulosic materials to include non-wood biomass, such as biological waste, recycled materials, and industrial waste. Examples of the non-wood raw materials and biowaste used to produce lignocellulosic materials include the following: cereal straw [16,17,18], flax and hemp shives [19,20], agriculture waste [21,22,23,24], and bamboo and miscanthus [25,26]. Wood-based raw materials, such as sawmill waste [27,28], recycled wood, and other byproducts of the wood industry, also constituted a separate group of raw materials analyzed. Particularly significant are construction and demolition waste, used furniture, and packaging waste, which constitute the most common stream of wood waste destined for recycling [29]. In an article by Wronka and Kowaluk [30], the possibility of recycling wood from window frames to produce particleboard was analyzed. The main objective of the study was to identify limiting factors for reusing wood through recycling window frames by conducting research under fully controlled conditions. The authors constructed new window frames from pine wood. The surfaces were finished using transparent and white coating systems, consisting of an acrylic primer, a base coat, and a top coat. After a year of conditioning, the frames were ground into wood particles. Their mass fraction in the board varied; according to the authors, the most promising results for MOR and MOE were obtained for boards containing more than 50% recycled wood. Unfortunately, most of the boards produced did not meet the minimum IB requirements, suggesting a need to modify either the gluing process or the raw material itself. The size of the particles was important in this case, especially in the core layer of the boards. As might be expected, the method of finishing the frames also influenced the properties of the boards. As noted, the clear varnish penetrated the wood more deeply, which resulted in better dimensional stability, reduced TS and decreased WA. In contrast, the white paint increased the bulk density of the wood particles due to its filler content. It is also significant that tests for formaldehyde emissions and total volatile organic compounds (TVOCs) showed that emission levels from particleboard made from window frame wood were comparable to those of the reference boards. As the authors state, this was likely due to the use of relatively new coatings that met current emission standards and the long seasoning period of the recycled material, which helped reduce formaldehyde emissions. In summary, the study conducted by Wronka and Kowaluk [30] indicates the high potential of wood derived from recycled window frames as a raw material for particleboard production, which supports the circular economy model. However, as rightly pointed out, the density and production process of the boards must be individually adjusted depending on the type of material used and the type of varnish coating previously applied to it.

In the context of recycled materials, Bartoszuk and Kowaluk (contribution 2) analyzed the possibility of using fibrous mat residues from upholstered furniture as a reinforcing filler in plywood. Due to its composition, this waste has limited recyclability and is therefore most often incinerated or landfilled. The aim of the study was to evaluate the feasibility of incorporating this type of waste into the structure of plywood in order to improve its properties, as well as the potential environmental benefits resulting from managing difficult-to-process waste. The study used a 200 g/m^2^ polyester nonwoven fabric, which was placed between layers of veneer in various configurations, ranging from its absence in the reference sample to variants with nonwoven fabric present in every layer of a five-layer birch plywood bond, glued with UF resin. It was found that placing the nonwoven fabric directly beneath the external veneers helped maintain and even increase the MOR, MOE, and IB values. For the SWR of plywood, the best results were obtained for the variant in which the nonwoven fabric was located on both sides of the middle veneer. It is also worth noting that incorporating nonwoven fabric into the outer layers of plywood had a positive effect on reducing its TS. According to the authors, in addition to the benefits of reinforcing the finished plywood, using this waste supports the principles of the circular economy and the Carbon Capture and Storage (CCS) policy, as the carbon contained in the nonwoven fabric remains “trapped” within the panel’s structure rather than being released into the atmosphere during waste incineration. Plywood reinforced in the proposed manner can serve as an ideal material for structural applications, particularly where high internal bond and screw withdrawal resistance are required, e.g., as structural sheathing in wooden frame buildings.

Research into utilizing waste in wood-based materials now encompasses not only post-consumer wood and industrial waste—primarily from the sawmill and furniture industries—but also a wide range of lignocellulosic raw materials and other bio-based materials, such as biowaste and marine biomass. The interest in these raw materials stems both from their wide availability and their potential applications. Non-wood biomass as well as forest and agricultural residues constitute some of the largest renewable resources available worldwide. Their use not only contributes to solving waste management problems but also to producing value-added materials and products, and aligns with the principles of a circular economy, supporting more sustainable management of natural resources [31]. For this reason, the research conducted by Papadopoulou et al. (contribution 3) is significant, as they assessed the upcycling potential of five types of biomass in producing particleboard, i.e., coffee bean husks, spent coffee grounds, thistle, *Sideritis* and dead leaves of *Posidonia oceanica*. UF resin and polymeric 4,4′-methylene diphenyl isocyanate (pMDI) were used as bonding agents, and the wood particle substitution rate was 30%. To understand the effect of using this biomass on the properties of the boards, the authors undertook detailed testing, demonstrating that the biomass used in the study contained cellulose, hemicellulose, and lignin in varying proportions, along with differing degrees of crystallinity. The highest thermal stability was observed for *Sideritis* and *Posidonia oceanica*. This type of biomass, particularly coffee husks and *Sideritis*, exhibits a higher buffering capacity than wood, which, according to the authors, may delay the curing of UF resin. As expected, substituting wood particles with this type of biomass resulted in a decrease in particleboard strength, although boards containing spent coffee grounds and *Posidonia* exhibited better dimensional stability as determined by their TS values. Additionally, regardless of the type of biomass, all produced board variants exhibited lower formaldehyde content than reference boards. The authors stated that integrating agricultural and marine biomass into the particleboard industry is feasible, although it requires optimization of the adhesive composition and the raw material pre-treatment processes.

Wronka et al. (contribution 4) proposed another approach to utilizing waste biomass in producing wood-based materials. The researchers used hazelnut shells as fillers in UF resin for plywood production. In addition to using the filler in its native form, the hazelnut shells underwent a delignification process to remove lignin and were carbonized at 600 °C. An analysis of the properties of the plywood produced using this filler showed that the addition of 10 wt% of raw hazelnut shell flour increased its MOR and MOE; in the case of chemically modified shells, optimal results were obtained at 5 wt%. In contrast, the addition of activated carbon (just 1% by weight) to the UF resin significantly improved the veneer bonding quality (more than threefold), reduced formaldehyde emissions, and reduced the WA of the plywood. These studies have therefore shown that hazelnut shells are a valuable bio-additive capable of replacing the traditionally used starch filler, which supports the circular economy, reduces the amount of biowaste, and lowers the carbon footprint of wood-based material production. The authors emphasize, however, that it is crucial to select the appropriate amount of filler, as an excessive amount negatively affects strength parameters.

### 2.3. Advanced Composite and Low-Emission Materials

Developing composite and low-emission engineering materials is currently one of the key areas of research in the field of modern construction technologies. Solutions that improve the performance of materials while making efficient use of available raw materials are of particular importance. One area of this research involves reinforcing wooden components, whose mechanical properties may be limited by natural defects in the wood, leading to reduced load-bearing capacity, stiffness, and increased deflection. To improve strength parameters and expand the potential for using lower-quality wood, various reinforcement techniques are employed for both solid and glued wood. Among the most commonly used solutions are metal reinforcements [32]. One important publication in this field is the article by Derkowski et al. [33] on the use of thermosetting adhesives in the process of reinforcing structural wooden beams with steel elements. As the researchers noted, the issue of joining steel and wood is well-known, but the epoxy and polyurethane resins commonly used for this purpose are expensive and difficult to apply. For this reason, they decided to test less expensive adhesives commonly used in the wood industry, which, thanks to their high pH, also protect steel from corrosion. To produce composite beams, they used pine lumber, smooth steel sheets, and ribbed steel bars. They tested four adhesive variants, including pure phenol-formaldehyde (PF) resin, pMDI, and two mixtures of PF and pMDI with the addition of recycled tire powder. To accelerate and increase the adhesive bond curing process efficiency, they used induction heating of the steel element located inside the pine wood sample. The researchers demonstrated that when using smooth steel sheet reinforcements, the highest joint strength was achieved with pMDI adhesive. However, in the case of ribbed steel bars, due to the voids between the wood and the bar’s core, the best solution proved to be a mixture of PF resin with pMDI and synthetic rubber, characterized by higher viscosity and increased reactivity than pure PF resin. Thus, the study demonstrated that traditional thermosetting adhesives can be effectively used to bond steel to wood, provided an adequate heat source is available for curing. Using tire waste in combination with commonly available industrial adhesives allows for the creation of cheaper and more environmentally friendly solutions in the production of materials intended primarily for timber construction.

A major trend in the development of modern and advanced composite materials is the use of lignocellulosic raw materials to modify polymeric materials. The growing interest in such solutions stems from the need to increase the share of renewable raw materials in engineering materials and to reduce the use of petrochemical-based plastics [34]. Siekierka et al. (contribution 5) also addressed the issue of developing sustainable composites for modern, energy-efficient construction. The researchers sought to improve the mechanical properties, thermal stability, and phase compatibility of the composites by adding fine and coarse coniferous wood flour to PVC plastisol (emulsion PVC with a plasticizer and stabilizer). To improve adhesion between the hydrophilic wood and the hydrophobic polymer, the flour was modified with silane and a nonionic surfactant. The authors found that composites containing fine silane-modified wood flour exhibited the best balance between stiffness, flexibility, and thermal resistance, as silanization ensured a stronger bond between the wood flour particles and the plastisol and made the material less susceptible to leaching under the influence of water. It was determined that the optimal wood flour content in the composite was below 15% by weight to ensure a uniform microstructure and stable properties. According to the researchers, the developed composites, thanks to their mechanical flexibility and thermal stability, can be used as protective and sealing layers in building exterior envelope systems, flexible coatings for precast concrete elements, and sealing elements in panel and expansion joints. The proposed solutions thus enable the creation of materials with a smaller carbon footprint, which is consistent with the concept of environmentally friendly and energy-efficient construction.

Developing composite materials with a limited environmental impact has also drawn researchers’ attention to the possibility of using biochar derived from wood waste as an additive in construction mortars. Biochar is one of the promising, sustainable materials being studied as a partial substitute for Portland cement in order to reduce its consumption and minimize the environmental impact of cement production [35]. Rykło et al. [36] evaluated the effect of adding biochar derived from wood chips at three pyrolysis temperatures—450 °C, 550 °C, and 700 °C—on the physicochemical, mechanical, and environmental properties of cement mortars. The authors showed that the pyrolysis temperature significantly affects the structure of biochar: biochar produced at 700 °C is characterized by the highest carbon content, alkaline pH, and high porosity, while that produced at 450 °C has more functional groups and better sorption properties. Its addition at 1–5% by weight improves the mortar’s parameters, allowing it to meet building code requirements for compressive strength and adhesion. However, dosages exceeding 10% may lead to structural weakening and reduced adhesion to concrete and expanded polystyrene (EPS). The researchers also analyzed the leachability of heavy metals and the emission of volatile organic compounds (VOCs), concluding that in most cases these levels remain within acceptable environmental standards. One of the most important conclusions of the study is that substituting traditional mineral fillers with biochar allows for a reduction in the carbon footprint of building materials by approx. 35%. The presented research results therefore confirm that biochar is a promising “carbon-negative” material that supports the goals of the circular economy and the climate neutrality strategy in the construction sector.

### 2.4. Advanced Technologies for Raw Material Modification and the Production of Wood-Based Materials

Developing modern material technologies also encompasses physical methods for modifying raw materials and using additive technologies in the design of lignocellulosic materials. An example of such research is the study presented by Wang et al. (contribution 6), which analyzed the effect of microwave–compression treatment on the mechanical properties, water resistance, and chemical composition of bamboo (*Phyllostachys heterocycla*). The researchers focused on how the initial moisture content of the material (10%, 30%, and 50%) altered the effectiveness of this process. The treatment process consisted of two stages: in the first stage, the bamboo was exposed to microwaves, which caused it to heat up rapidly and softened the lignin, and was then compressed in a press to achieve a 30% compression ratio. Bamboo with low moisture content, around 10%, exhibited the highest strength, including the highest MOR and MOE values. Higher bamboo moisture content (30% and 50%) resulted in a significant decrease in these parameters, which the authors attributed to structural damage caused by rapidly evaporating water and high internal pressure. Nevertheless, bamboo with higher moisture content exhibited better dimensional stability and less swelling after soaking in water, due to processing under such conditions promoting the cross-linking of cellulose, hemicellulose, and lignin. Furthermore, it was found that higher moisture content promoted an increase in the cellulose crystallinity degree, while the applied treatment led to densification of the fiber structure. At the same time, it was demonstrated that excessively high moisture content in bamboo can cause damage to its microstructure in the form of cell wall cracks. The authors therefore demonstrated that combining microwaves and pressing is an effective and environmentally friendly method for strengthening bamboo by allowing for the improvement of its strength parameters and dimensional stability, which may find application in civil engineering, as well as in producing furniture and flooring panels.

In parallel with research into new materials, additive technologies (AM) represent a significant development trend in the construction industry, offering new possibilities for designing and manufacturing components while minimizing material consumption. 3D printing is the most commonly used term for these technologies, particularly in the context of their industrial and construction applications. Additive manufacturing (AM) is an advanced manufacturing technology in which three-dimensional components are created by successively applying layers of material according to a digital model developed using CAD (Computer-Aided Design) software [37]. Tanikella et al. [38] proposed a very interesting solution in this area, aiming to develop sustainable composite materials for 3D printing that serve as a low-cost and environmentally friendly alternative to traditional building materials. It should be emphasized that 3D printing is currently viewed as one of the most promising areas of development for building materials and structures, enabling the optimization of production processes, a reduction in material consumption, and greater design freedom. The authors, utilizing byproducts from the wood and agricultural industries (wood fibers, hemp hurd, and hemp fiber), developed and characterized wood–sodium silicate composites specifically intended for additive manufacturing. Composites differing in the content, type, and size of lignocellulosic particles, as well as in sodium silicate content, were tested. It was determined that finer lignocellulosic raw material particles ensure better compaction and greater strength. In turn, rheology and printability depended mainly on the sodium silicate content. Mixtures with low silicate (liquid) content were too “dry,” which resulted in high demands on the extruder’s motor power and problems with print fluidity. It is significant that this is the first time it has been demonstrated that hemp shives effectively reinforce biocomposites intended for 3D printing, improving their density and mechanical properties. The authors conducted a cost analysis, comparing the developed materials to the popular PLA plastic. The formula consisting of wood fiber pellets, 55% sodium silicate, and 10% hemp shives demonstrated the most favorable strength-to-price ratio (73.0 MPa/$) and good processing properties.

## 3. Conclusions

The research results presented in this Special Issue focus on developing modern wood-based materials and systematically expanding knowledge regarding the physicochemical properties of alternative biomass, which forms the foundation of modern, sustainable construction. The high potential of previously overlooked raw materials (aspen, hazel, young wood) has been demonstrated, as well as the effectiveness of utilizing various types of waste (window frames, nut shells, coffee grounds, upholstery fabrics) as full-value substitutes for traditional wood. Through using innovative technologies, such as additive technologies, chemical or physical modifications of the raw material, or the addition of “bio-additives,” the foundations have been laid to produce durable, low-carbon composites with optimized structural parameters and reduced emissions.

## Data Availability

Not applicable.
